# Bacterial nanocellulose: engineering, production, and applications

**DOI:** 10.1080/21655979.2021.2009753

**Published:** 2021-12-02

**Authors:** Reshmy R, Eapen Philip, Deepa Thomas, Aravind Madhavan, Raveendran Sindhu, Parameswaran Binod, Sunita Varjani, Mukesh Kumar Awasthi, Ashok Pandey

**Affiliations:** a Post Graduate and Research Department of Chemistry, Bishop Moore College, Mavelikara, India; b Rajiv Gandhi Center for Biotechnology, Thiruvananthapuram, India; c Microbial Processes and Technology Division, CSIR-National Institute for Interdisciplinary Science and Technology (CSIR-NIIST), Trivandrum, India; d Paryavaran Bhavan, Gujarat Pollution Control Board, Gandhinagar, India; e College of Natural Resources and Environment, Northwest A & F University, Yangling, China; f Centre for Energy and Environmental Sustainability, Lucknow, India; g Centre for Innovation and Translational Research, CSIR- Indian Institute for Toxicology Research, Lucknow, India

**Keywords:** Bacterial nanocellulose (BNC), biosynthesis, genetic modification, static fermentation

## Abstract

Bacterial nanocellulose (BNC) has been emerging as a biomaterial of considerable significance in a number of industrial sectors because of its remarkable physico-chemical and biological characteristics. High capital expenses, manufacturing costs, and a paucity of some well-scalable methods, all of which lead to low BNC output in commercial scale, are major barriers that must be addressed. Advances in production methods, including bioreactor technologies, static intermittent, and semi-continuous fed batch technologies, and innovative outlay substrates, may be able to overcome the challenges to BNC production at the industrial scale. The novelty of this review is that it highlights genetic modification possibilities in BNC production to overcome existing impediments and open up viable routes for large-scale production, suitable for real-world applications. This review focuses on various production routes of BNC, its properties, and applications, especially the major advancement in food, personal care, biomedical and electronic industries.

## 1. Introduction

**Figure 1.**
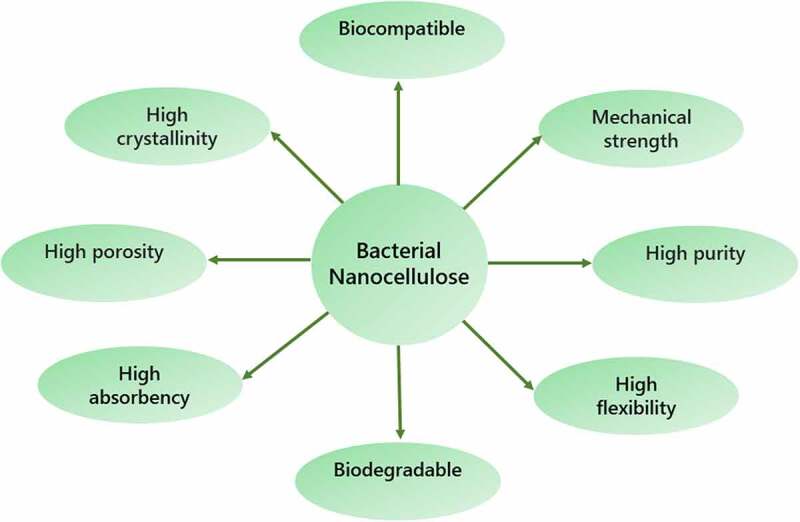
Characteristics of bacterial nanocellulose

Bioprocessing techniques generally employ metabolically engineered microorganisms such as *Escherichia coli* or *Saccharomyces cerevisiae* to convert biomass into value-added biomaterials such as biofuels, platform chemicals, bioplastics, and extracellular polymeric substances [1,2]. Researchers utilize a diverse range of biomasses for numerous environmental applications on a regular basis. Today, scientists have shifted their focus to the development of more active and safe materials that can be utilized in a variety of applications owing to the limits of both synthetic and natural materials. BNC is a potential biopolymer produced as an extracellular macromolecule of β-D-glucopyranose by specific types of bacteria [3]. Despite the fact that it contains 99% water, BNC has excellent mechanical characteristics [4,5]. The specific characteristics of BNC are depicted in Figure 1.

BNC is extremely suitable for cellular adhesion and immobilization because of its water-holding capability and nanostructured shape, which is comparable to that of the cell protein, collagen. BNC, in addition to its many unique characteristics, falls under the category of generally regarded as safe (GRAS) materials, making it suitable for a wide range of applications. Mainly, BNC biocomposites are utilized in wound healing, drug delivery, artificial blood vessel, and tissue regeneration (Biofill is an example) [6]. BNC might be more viable in the biomedical industry if the high cost of production can be reduced by applying techniques that utilize sophisticated multifunctional culture medium and moving bioprocessing to high-efficiency bioreactors.

According to a global forecast analysis, the BNC market would expand three times faster in the next 5 years. The substrate selection, biosynthesis mechanisms, genetic modifications, instability challenges, and feasibility of production techniques are the major criteria to optimize for cost-effective future BNC market. The biosynthetic process is gaining popularity as it leads in the self-assembly of secreted fibrils with distinct physiochemical properties. Biosynthetic methods can provide excellent purity for BNC since it is devoid of pectin, lignin, and hemicellulose. Thus, tedious purification processes can be avoided. The biosynthetic pathways have revealed the identification of possible microorganisms involved in BNC production [7]. The optimal degree of biosynthetic pathway enzyme expression should be assessed to avoid excessive metabolic burden induced by overexpression of the system, as well as insufficient supply of critical precursors for downstream biosynthesis. For instance, sugar-rich biowaste such as fruit and vegetable waste, sugar cane bagasse, wood processing residues, and so on with cheaper availability is attaining immense significance as substrates for biosynthesis. Thus, cost-effective BNC production using innovative feedstocks, as well as scaling up through better bioprocess techniques, necessitates a more targeted researches in order to completely exploit BNC for commercial applications.

The scaling up process of the BNC production has many hurdles including nature of target metabolite, longer biosynthesis duration (5–20 days), low yield (8 g/L) [8]. Upgrading fermentation technology is one of the potential avenues for improving the yield of the BNC production. However, because the microbial producers of BNC are strict aerobes, a concern arises regarding how to manage culture, whether it is static or dynamic. Kralisch et al. combined static and agitated cultivation methods to develop a semi-continuous culture strategy by using a horizontal lift reactor for harvesting biofilms of BNC. This approach was utilized in process scale-up for decreasing cost of production considerably [9]. Characteristics of BNC for implementation in different applications are depicted in Table 1.

**Table 1.** Characteristics of bacterial nanocellulose for implementation in various applications

Sl No.	Properties	Implementation	References
1	Mechanical strength	BNC’s impressive mechanical characteristics make it ideal for use as a load bearing material in a variety of applications, including food packaging, and medicinal implants.	[6,10]
2	Withstand extremely high temperature	Sterilization using Gamma rays or steam is simple; it’s ideal for packing sterile equipment.	[3]
3	Absorbency and resistance to fiber lift	For paper based dressings	[3]
4	Biocompatibility- nontoxic to human cells	BNC-based implants, wound healing dressings, and personal care products can all benefit from this property.	[108]
5	Wet strength – capability to withstand extremely high temperature	As labeling adhesive for clinical samples, such as blood while being kept in deep freezers, which can withstand extremely low temperatures.	[11,12]
6	Application orient processibility	Enhanced mechanical, and barrier property, along with oil absorbency, air permeability, and antimicrobial properties upon functionalization open up easy molding to different shapes for wider applications.	[13,14]
7	Inertness – to avoid any reactions between packed products during storage	For packing food items, medicines, and surgical devices	[15,16]
8	Biodegradability	Scaffolds should disintegrate within the body after the primary extracellular matrix development starts, thus this is very useful when building them.	[17,18]

The present review focus on various aspects of biosynthesis of BNC, effective substrate utilization strategies, current status, challenges, and future prospects of application of BNC in biomedical, food packaging, cosmetics, and electronics are discussed with special emphasis to cost-effective strategies.

## 2. Biosynthesis of bacterial nanocellulose

BNC is an extracellular biopolymer that forms a three-dimensional network at the air-liquid culture medium interface. A. J. Brown first reported bacterial cellulose in 1886, when he observed *Acetobacter xylinum* cells producing cellulose with glucose and oxygen [19]. Acetobacter has been renamed as *Komagataeibacter*, and it is one of the best rod shaped Gram negative acetic bacterial species. The present researches suggest a special focus on bacterial strains of this species that develop into a model entity for molecular, biochemical and genetic studies because of the intriguing phenotype and high cellulose output [20]. Other notable BNC producers include *Dickeyadadantii, Rhizobium leguminosarum, Agrobacterium tumefaciens, Salmonella enterica, Escherichia coli*, and *Pseudomonas putida* [21]. The development of genetically altered BNC-producing strains is one of the most intriguing prospective ways for improving BNC production while also lowering costs [22].

### 2.1. Molecular mechanisms involved in biosynthesis

BNC synthesis is a multi-stage, sophisticated, accurate, and tightly regulated process. It involves a vast number of genes that code for particular enzymes and regulatory proteins found in catalytic complexes. This is a multi-step method that involves enzymes and complexes of regulatory and catalytic proteins, all of which are precisely and carefully regulated. Cellulose synthase has a significant role in the production of BNC, working in tandem with endo β-1,4-glucanase, the complementing factor, and β-glucosidase. Their genes are found in the flanking areas of operons. BNC biosynthesis is associated with a number of metabolic processes, including the phosphogluconate pathway and the Embden Meyerhof Parnas route, gluconeogenesis, and the Krebs cycle.

**Figure 2.**
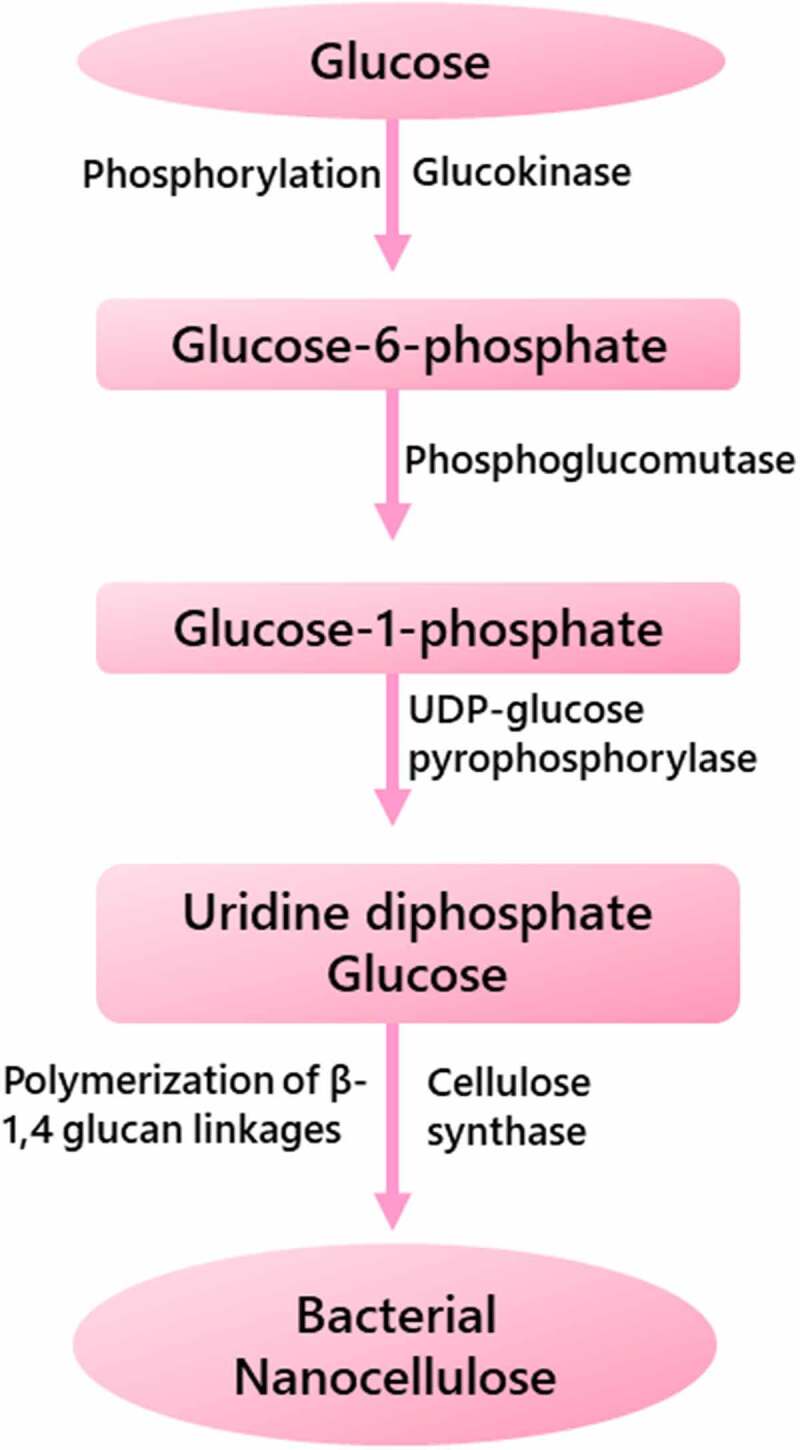
Schematic representation of biosynthesis pathways of bacterial nanocellulose production

The phosphogluconate cycle includes the oxidation of carbohydrate, while the Krebs cycle involves the oxidation of acetate-derived carbohydrates, proteins and fats [23]. The biosynthetic pathways of BNC production involves four main enzymatic stages as shown in Figure 2: (i) glucose into glucose-6-phosphate conversion through phosphorylation using *glucokinase*; (ii) glucose-1-phosphate isomer production from glucose-6-phosphate by *phosphoglucomutase*; (iii) uridine diphosphate glucose (UDP-Glc) production from glucose-1-phosphate using *UDP-glucose pyrophosphorylase*; and (iv) finally, cellulose production by the polymerization of UDP-glucose through β-1,4 glucan linkages in presence of cellulose synthase (Bcs) [24].The pathways and methods of UDP-Glc production are generally understood, however the glucose polymerization mechanism in unbranched and long chains remain unknown.

Bcs is made up of four different protein subunits: BcsA, BcsB, BcsC, and BcsD. The genes that code for these distinct proteins are all controlled by a single promoter, forming the ‘bacterial cellulose synthase’ operon (bcs). The catalytic subunit that forms BNC and produces the transmembrane pore through the inner membrane is the BcsA membrane protein [25]. BcsB is a periplasmic protein that interacts with BcsA and is linked to the inner membrane by a single transmembrane helix (TM). BcsB’s function is thought to be to use two carbohydrate binding domains to lead the polymer through the periplasm to the outer membrane. BcsB is essential for BcsA’s catalytic activity in all circumstances because their contact allows the TM region of BcsA to be stabilized, resulting in catalytically active synthase [26].

The BcsC protein has a beta barrel inside the outer membrane, which is preceded by a periplasmic domain containing a tetratricopeptide repetition, indicating that it is involved in the complex’s assembly. By generating a pore at the outer membrane, BcsC would make it easier for periplasmic cellulose to exit the cell [27]. BcsD is a periplasmic protein that is not required for cellulose synthase to function. The BcsD protein would then play a role in cellulose sub-fiber extrusion or crystallization. Nonetheless, the BcsD protein’s exact function and mode of action are unknown [28].

### 2.2. Genetic modification possibilities

Despite intensive and long-term research that takes into account changes in culturing environments, it is quiet hard to perfectly regulate the biosynthesis and characteristics of cellulose generated by *Komagataeibacter* species. A key impediment to larger-scale productions of BNC from *Komagataeibacter* strains is the absence of economic feasibility owing to their low productivity. One of the main disadvantages is that the nutritional requirements and production efficiency of various *Komagataeibacter* strains vary greatly, as well as the spontaneous formation of unstable Cell-mutants in agitated cultures, resulting in the consumption of nutrients for cell growth and multiplication without cellulose production [22].

Extensive genetic testing is required to achieve this goal, which will reveal the molecular links between proteins engaged directly and indirectly in BNC synthesis. Efforts to boost the competence of BNC production or create structural modifications in the BNC network, thereby giving these nanomaterial new capabilities, have been the focus of genetic engineering of *Komagataeibacter* strains thus far. The expression of a unfamiliar gene or the destruction of a gene were among the genetic alterations [29].

Researchers have used homologous recombination, heterologous gene expression, and innovative techniques such as Clustered Regularly Interspaced Short Palindromic Repeats (CRISPR) to change the genomes of BNC-generating bacteria, and the gene sequencing of a few *Komagataeibacter* strains has provided this research direction a much-needed boost. Electroporation, which involves the development of some uneven pores in the surface of cells under the electric field, is the most often employed approach for altering *Komagataeibacter*. Pores allow macromolecules from the intercellular space to enter the cells [30,31].

The genes phosphogluconate dehydrogenase and glucose 6-phosphate isomerase were predicted to be new targets of overexpression for increased BNC synthesis in studies because they displayed a constructive correlation with BNC generation during arbitrary sampling for a total of 16 reactions from the glycolysis and pentose phosphate pathways. Conjugation with *E. coli* cells is another technique of transformation identified for *Komagataeibacter xylinus* (*K. xylinus*) [32].

**Figure 3.**
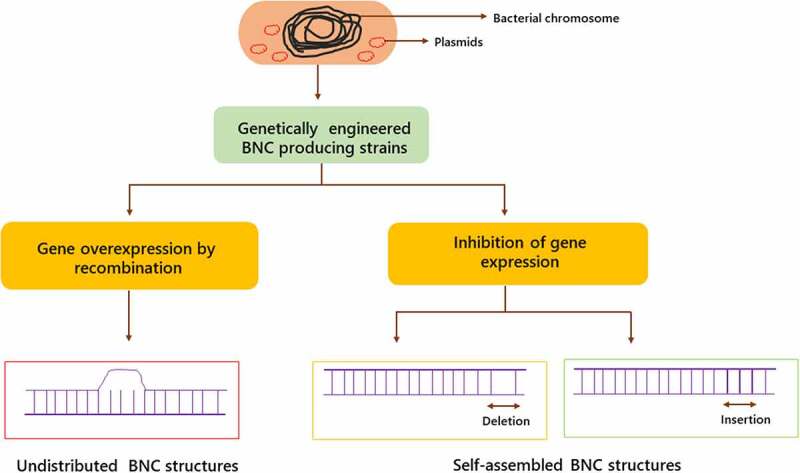
Genetic engineering stages in the production of bacterial nanocellulose

In *K. xylinus* species, replicating plasmids such as pTI99, pLBT, pBAV1C, pBBR122, and pSA19 shuttle plasmids are utilized as vectors for gene expression or overexpression [33]. However, a case of transposon mutagenesis employing the mini-Tn10 transposon has been recorded as well. When the purpose of the transformation is to interrupt one of the bacterial chromosome’s genes, the matching sequence is incorporated into *K. xylinus* cells using a non-replicating plasmid, such as pACYC184, BPR2001, pKE23 or pET-14b [34].These sequence contains several genes of interest, which is interrupted by an antibiotic resistance gene. Homologous recombination leads to the generation of cells with antibiotic resistance and the injected plasmid does not multiply in *K. xylinus* cells. As a result of the disruption, gene expressions are inhibited in these cells [35]. Genetic engineering steps in the production of BNC are represented in Figure 3.

#### 2.2.1. Inhibition of gene expression

In 1990, during research on the Bcs operon and its genes, the method of disrupting gene expression was applied for the first time. The plasmid pACYC184 was utilized for this, and the bBcsD gene sequence was cloned into it before the ampicillin resistance gene sequence was halted. The cellulose synthesis efficiency of obtained mutants was lowered by roughly 40%, leading to the conclusion that the bcsD gene is necessary for optimal biopolymer synthesis efficiency in vivo [28]. However, Saxena and coworkers discovered that the broken bcsD mutant formed both cellulose I and cellulose II alomorphs, indicating that the bcsD genes are taking part in cellulose crystallization. The BcsD protein is thought to be part of the pore organization in the linear terminal complex and the mutant cells indicated irregularities in the alignment of complex on BcsD mutant cells, whereas the constituents within each linear terminal complex seemed to be oriented appropriately and operated similarly to wild-type cells [27]. Moreover, Mehta et al. found that BcsD assists in the right alignment of the linear terminal complex along the cell’s longitudinal axis, implying that this protein plays a function in the last phase of cellulose hierarchical assembly, leading to highly proficient cellulose production [36]. Bae et al. reported genetic engineering of the *K. xylinus* BPR2001 strain to evaluate BNC constructed by mutants with dgc1 gene disruption to the wild-type strain’s BNC. Because the dgc1 gene is involved in the stimulation of BNC biosynthesis via c-diGMP, its interruption should result in lower output. However, it was discovered that knocking down the dgc1 gene had little influence on BNC synthesis but had a significant effect on its structure. The dgc1 mutant produced BNC membranes with small scattered fibers that did not form a typical hydrated membrane [37].

The aceP gene, which codes for the glycosyltransferase engaged in making of the extracellular branched heteropolysaccharide acetan, has also been disrupted. *Komagataeibacter xylinus* produces this water-soluble polysaccharide from UDP-Glucose. The CHE5 disruptive mutant of *K. xylinus* produced acetan with a different structure but had the same yield [35]. Furthermore, Ishida et al. disrupted the aceA gene in *K. xylinus* BPR2001, which encoded with the b-glucosyltransferase for the initiation of acetan biosynthetic pathway. This mutant generated more BNC under shaking conditions compared to the wild-type strains, despite the fact that suppression of acetan synthesis was anticipated to enhance the concentration of UDP-Glucose. The EP1 mutant’s culture media developed a heterogeneous suspension with huge flocs generated by cell aggregates and BNC after two days. Additionally, incorporating water-soluble polysaccharides like acetan or agar to the culture media increased the number of free cells by dispersing the material. The lack of acetan decreases the viscosity of the culture fluid and enhances cell and BNC aggregation, lowering BNC output [38].

The disruption of the ghd gene sequence in *K. xylinus* BPR2001 cells, which codes for glucose dehydrogenase (GDH), is another example. The extracellular transformation of glucose to gluconic acid, which does no longer exist as a substrate for BNC synthesis, is carried out by the mutant strain’s GDH activity. In the pT7-Blue T-Vector plasmid vector, the cat-1 chloramphenicol acetyltransferase gene was substituted with the GDH coding gene. The mutant showed any GDH activity, but it did have a higher cellulose synthesis efficiency [39]. In comparison to the wild-type strain, the mutant (GDH-KO) *K. xylinus* BCRC 12334 with the gdh gene disrupted generated more BNC. The improved mass transfer of O_2_ and nutrients in shaking culture has resulted in a considerable rise in BNC production. It was also shown that the GDH-KO mutant may generate BNC from glucose without creating gluconic acid as a byproduct [34].

#### 2.2.2. Gene overexpression

Mangayil et al. reported the overexpression of the bcsA genes, bcsAB, and the entire cellulose synthase operon (bcsABCD) in *K. xylinus* DSM 2325 effectively. Since there were any substantial variations in the growth between mutants and wild-type strains, mutants produced two to four times more BNC. The bcsABCD overexpression mutant had the highest efficiency [33]. CMCax gene overexpression in *K. hansenii* ATCC 23769 improves the output of BNC, and adding CMCax protein to the culture medium enhances the formation of cellulose. The expression of the cmcax gene can be used to control BNC synthesis [40]. Nakai et al. examined CMCax’s impact on BNC biosynthesis (2013) The BPR 2001 mutant of *K. xylinus* with the cmcax gene disruption generated less BNC than the wild-type strain [41]. Furthermore, it was discovered that the mutant only formed cellulose II, implying that CMCax influences cellulose crystallinity. Kawano et al. used electron microscopy to show that CMCax can influence the cellulose fiber structure. In addition, cellulose fibers released by the ATCC 23769 *K. hansenii* strain overexpressing the cmcax gene had a more undisturbed structure than wild-type strains [40].

### 2.3. Genetic instability challenges

The vast variation in dietary needs and process efficiency across *Komagataeibacter* strains is a significant barrier to the industry’s adoption of the BNC biosynthesis method. The genetic fragility of some BNC producing strains, spontaneously create cellulose-negative mutants when agitated, limiting total productivity. The non-Newtonian behavior of BNC in the fermentation medium, which causes a large rise in viscosity with a subsequent decrease in homogeneity and oxygen distribution in the medium, is another factor that may have a detrimental impact on BNC productivity under agitation.

Hestrin and Schramm discovered cellulose-negative mutants in shaken cultures for the first time in 1954 [42]. It has been demonstrated that agitated liquid culture with homogenous aeration promotes the spontaneous emergence of cellulose-negative mutants, which eventually become dominant. Despite the extensive investigation into the formation of cellulose-negative mutants, the chemical basis for this occurrence has yet to be discovered. The search for the best culture conditions for reducing the generation of cellulose-negative mutants led to the discovery of tactics such as substituting glucose with fructose or adding the glucose medium with ethanol, as well as choosing the right agitation speed [43]. Since these strains frequently carry numerous plasmids of varying sizes, it was assumed that plasmids from *K. xylinus* might possess a role in BNC biosynthesis. Despite the fact that cellulose positive and cellulose-negative cells typically have variable plasmid profiles, several BNC-synthesizing strains have been discovered without plasmids [44]. IS1031 insertion elements have also been linked to *K. xylinus* producing unstable cellulose [45]. Existence of IS elements in the middle of genes, interrupting the coding sequence and deactivating the gene’s expression [46]. The presence of the IS1031 insertion element has been linked to the cellulose-negative morphotype. The cellulose-negative morphotype lacks two essential enzymes in the cellulose biosynthesis pathway: phosphoglucomutase and glucose-1-phosphate urydilotransferase [47].

## 3. Different production techniques for bacterial nanocellulose biosynthesis

BNC biosynthesis is a well-organized and strictly controlled process and has two stages: first, the formation of 1,4-glucan linkages, and subsequently the assembly and cellulose crystallization. In a brief, the process starts with the carbon source such as glucose and fructose being transported into the cell, where the cellulose precursor UDPG is produced. Bcs then polymerizes glucose from UDPG into 1,4-glucan strands. Finally, cellulose chains are secreted as sub-fibrils through pores in the cell membrane, then combined into ribbons in a 3D nanofiber network supported by hydrogen bonds.BNC is the product of an oxidative fermentation process that involves the oxidation of sugars and organic acids in synthetic or non-synthetic sugar and nitrogen-rich media at temperatures ranging from 25 to 30°C and pH 4.5 to 7.5 [48].

**Figure 4.**
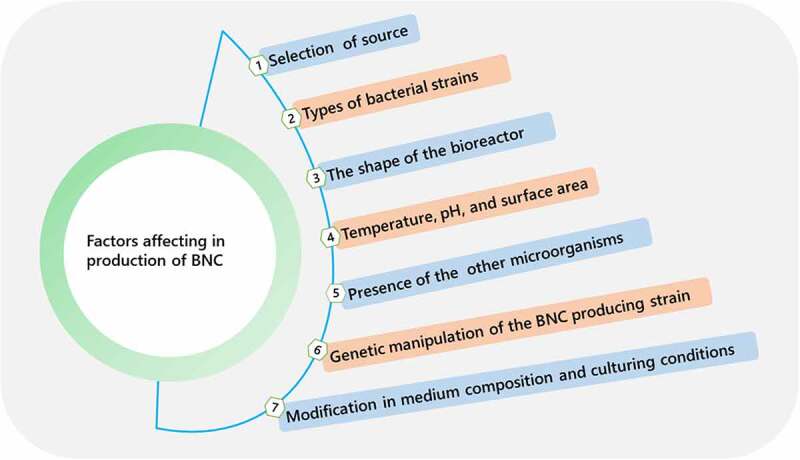
Different factors affecting biosynthesis of bacterial nanocellulose

BNC produced by various bacteria under a variety of culture conditions has been demonstrated to have a variety of structural properties that define its unique physicochemical characteristics and morphologies, making it appropriate for numerous biomedical and industrial applications [49]. Various factors affecting biosynthetic production of BNC are illustrated in Figure 4. Static, agitated, and bioreactor-based bacterial culturing are currently the three methods for cellulose production. Macro morphology, which possesses a variety of physicochemical features as a result of specific types of cellulose assemblies into nano- and micro-structures, differs significantly between these procedures. As a result, the choice of a production technique will be determined by the applications of BNC with unique properties.

The culture atmosphere, which includes bacteria strain, nutrients, oxygen delivery and pH is also important and has an impact on BNC characteristics [50]. When compared to cellulose produced by static culture, agitated culture cellulose has a lower mechanical strength. Furthermore, agitated cultures give less yields than static cultures and have a larger risk of microbe mutations, which could impact BNC production. Static culture, on the other hand, necessitates a greater cultivation area and much culture time [51]. When analyzing the characteristics required producing BNC, there are a few things to bear in mind. The culture medium’s composition has a direct impact on the effectiveness of the biotechnological process, thus it is crucial for the generation of any bio product, including BNC [52]. The different production methods and their advantages and disadvantages are provided in Table 2.

**Table 2.** Major production techniques for bacterial nanocellulose production

Production method	Description	Advantage	Disadvantage	References
Static fermentation	Media components are assorted at the starting; BNC production takes place in the vessel at the air-liquid interface.	Simple technique; No need of sophisticated instruments.	Time consuming process; Monitoring of fermentation condition is difficult; BNC yield affected by two forms of cellulose namely pellicle or reticulated slurry.	[53,83]
Agitated fermentation	Reciprocal agitation at 90–100 rpm; Stirring facilitates rapid cell grow.	Feasible for industrial scale manufacture; Easy to overcome diffusion; Operator friendly.	BNC formed as as irregular spheres not as pellicle; Culture mutation possibilities due to agitation at high rpm; Low yield; Culture instability problems.	[72,84]
Static intermittent fed-batch technique	Specific fresh media help to grow pellicle in huge rate in irregular time intervals.	Simple process; Highly improved production; Large scale productivity.	No proper monitoring of fermentation conditions; Cellulose separated as pellicle, or occasionally as reticulated slurry.	[54,107]
Bioreactor assisted production (Rotary disc and Air lift reactors)	New concept of Rotating Biological Contactor; The organisms were soaked in nutritional media and then exposed to air using discs.	Elevated productivity; Less work-up; Viable to scale-up.	High productivity only if culture media and conditions are properly maintained.	[55–57]
Cell-free extract technique	Cell lysis liberates most of the enzymes necessary for BNC synthesis straight to the medium.	Easy process; Large scale feasibility; Enhanced yield since culture processibility need short time.	No proper control on fermentation parameter.	[58,59]

A carbon source, nitrogen supply, and sulfur, phosphorus, magnesium and potassium salts essential for microbe development must all be present in the fermentation medium at a minimum. Chemical agents and the physical influence of chemicals existing during cellulose manufacturing can affect the synthesis of cellulose. As a result, the culture medium utilized may have an impact on aspects including yield, morphology, structure, and physical qualities, regardless of the manner of production [60].

### 3.1. Static fermentation method

A variety of methods for BNC creation were used, ranging from batch and fed-batch cultivation to continuous cultivation methods using standard bioprocesses, such as bioreactors. The production process has a direct impact on the supramolecular structure of BNC as well as its mechanical and physical properties; hence the production strategy chosen is determined by the end usage of BNC. The standard type of BNC cultivation method is static production, which produces a highly uniform supramolecular BNC structure. This culture technique is favorable for the generation of flat BNC with high-purity structures and qualities for its intended use. It makes pellicles with a lamellar structure and minimal branching, which are used in wound dressing.

The static culture method is a well-known and widely used method for producing BNC. It is a widely utilized technique for the creation of BNC at the lab scale since it is a comparatively easy process with a low shear force environment. A culture medium with a pH of 4.5 to 6.5 is placed into various shapes and sizes of containers and incubated. The static process produces BNC that takes the shape of the container that holds the nutrient medium.This method has been shown to produce the most homogeneous supramolecular BNC structure resulting in highly stable material based on various studies. As a result, this method of cultivation is considered as the ideal method of manufacturing flat BNC with excellent and reproducible structures and properties for commercial applications. The dessert natade coco is well established in the Asian food market as one of the first purchasable products based on BNC. It’s usually made using static cultivation, which involves skimming the product as sheets from the culture medium.

Kralisch et al. devised a method for producing semi-continuous planar BNC fleeces, and foils with enviable properties. A horizontal lift reactor was used in this unique technique to continually manufacture BNC with a homogeneous, 3D structure identical to BNC produced in a static environment. Smooth extraction of the right size BNC is achieved thanks to this superior mix of continuous processing and almost static cultivation. BNC was collected using an extractor mechanism that did not damage the medium or the resulting BNC sheet [9].

BNCs grown in a static culture environment have a consistent film shape. Hestrin and Schramm first described the culture medium commonly utilized for the generation of BNC in 1954, independent of whether static or stirring culture is used. 2.0% glucose (the primary carbon source), 0.50% yeast extract, 0.50% peptone, 0.27% anhydrous Na_2_HPO_4_, and 0.15% C₆H₈O₇.H_2_O make up this medium. This medium, on the other hand, can raise the biopolymer’s ultimate production cost and is regarded unsuitable for commercial BNC manufacturing due to its high cost [48,61]. Because of the expensive synthetic medium and low yield rates of BNC synthesis, this approach is unsuited for large-scale industrial manufacturing. Inadequate oxygenation of static cultures of exclusively aerobic bacteria is one of the key issues [62]. Studies on the viability of using fruit juices and/or agricultural and industrial waste as carbon and nutrient sources have been conducted in an attempt to address some of these difficulties. The morphology, microstructure, and other characteristics that result BNC’s inherent characteristics are essentially identical to those of BNC obtained from standard culture.

In the meanwhile, these research have shown the possibility for waste items to be used in environmentally acceptable and sustainable ways BNC manufacture at a low cost [63,64]. To lower the expense of the culture media in stirred culture, Jung et al. used corn steep liquor and molasses. The researchers also evaluate the potential of organic acids for boosting BNC synthesis. As a result of the presence of acetic acid, the maximum yield was obtained. Furthermore, the BNC generated from molasses and complex media had identical FT-IR spectra, demonstrating that the microbes could metabolize various carbon sources [65].

Li et al. investigated the utilization of candied jujube wastewater (WWCJ) in the static culture of *G. xylinus* CGMCC 2955 to yield BNC. WWCJ included primarily glucan, glucose, and extremely less quantities of other carbohydrates, making it a promising carbon source to produce BNC. WWCJ medium with sodium dihydrogen phosphate, ammonium citrate and calcium carbonate, WWCJ without ammonium citrate, and WWCJ hydrolyzate at 80°C were all used. In all of the settings studied, the bioprocess required 6 days and boosted BNC production. The WWCJ without ammonium citrate media, on the other hand, produced the lowest BNC output, suggesting that ammonium citrate may be an important element in BNC formation [66].

Dubey et al. used static intermittent fed batch technology to boost BNC synthesis from the *Komagataeibacter europaeus* SGP37 strain using sweet lime pulp waste. As the pellicle thickens and covers the entire surface area of the vessel in static BNC production, the oxygen and nutrition availability to the microorganisms is reduced. The yield of BNC is reduced as a result of this situation. To get over this limitation, sporadically supplying fresh media directly over the produced BNC pellicle was used to encounter nutrient delivery and the high oxygen demand [67].

Huang et al. were the pioneers to employ lipid fermentation effluent as a substrate for *G. xylinus* BNC extraction. The chemical oxygen demand (COD) value of lipid fermentation wastewater was 25.59 mg/L, indicating a low BNC output. According to the study, hydrolyzing extracellular polysaccharides in lipid fermentation wastewater may make the wastewater more biodegradable and increase BNC production. Furthermore, the structure of BNC was very little affected by the lipid fermentation wastewater environment [61].

Several groups have experimented with different carbon sources in order to boost BNC yields while lowering manufacturing costs. Low-sugar carbon sources have yielded intriguing results. Although using alternate carbon sources can help increase BNC production, environmental parameters such as pH and temperature must also be controlled. Temperature influences microorganism development and, as a result, cellulose production. The dissolved oxygen concentration in the culture medium, in addition to pH and temperature, is a significant component that can influence cellulose synthesis [68,69].

### 3.2. Agitated fermentation method

In static culture systems, the two main issues are high costs and limited production rates. The adoption of an agitated/ shaking culture has been offered as a solution to these issues. The distribution of oxygen is acknowledged to be a key problem of the static culture method, as it is directly linked to the creation of BNC. Excess oxygen supply, on the other hand, has been proven to reduce BNC formation. An agitated culture was created with the goal of increasing or optimizing oxygen delivery to bacteria during culture.

The culture duration, spinning speed and nature of additive in the culture medium all influence the size and form of the BNC. The incessant shear force during agitated culture is the first factor that generates a spherical structure during rotationally agitated cultivation [70]. Another element that influences the size and quantity of spherical BNC is the duration of culture. The quantity and size of sphere-like BNC were also observed to be affected by the concentration of bacteria. More BNC spheres can be produced if the bacteria concentration is higher [71].

The agitated approach has a number of problems, including bacterial strain instability, non-Newtonian behavior during BNC mixing, and a large shear force. BNC generated by agitated culture, on the other hand, shows some microstructural and property alterations including a low degree of polymerization, low crystallinity index, and poor mechanical characteristics [72,73]. In a static culture, a BNC membrane can form on the nutrient’s air liquid interface, while in an agitated culture, BNC is synthesized from the particle’s center and subsequently spreads outwards [74]. As a result, the microstructure of sphere-like BNC has a layered structure. Within the multilayer structure, BNC fibers that are significantly denser and contain bacterial strains have been discovered. Despite these issues, some study suggests that agitated culture may be the best strategy for large-scale production at a low cost [71].

Czaja et al. employed agitated fermentation to produce BNC using the ATCC 53582 *G. xylinus* strain that produced BNC in the form of big, distinctive spheres. It was found that cellulose generated in stationary culture had uniaxial oriented ribbons, but cellulose synthesized in agitated conditions had a disordered, bent, and overlapping ribbon-like shape [75]. In an agitated culture, rotation speed is also significant in the production of sphere-like BC. It’s difficult to identify sphere-like BNC particles when the rotating speed is less than 100 rpm; instead, irregular shapes of synthesized BNC have been reported [74].

Cheng et al. analyzed the efficiency of various plastic composite supports (PCS) in a submerged fermentation process to improve the material qualities and outcome of BNC. The goal of employing PCSs was to create BNC biofilms that grew on a solid platform and connected microbes as a natural type of cell immobilization. PCSs are made up of polypropylene and a variety of nutritious ingredients namely bovine albumin, soybean flour, red blood cells, yeast extract, peptone and minerals that are gently released into the media during fermentation, assisting in the development of BNC [69].

### 3.3. Industrial scale production

With the growing commercial interest in BNC, a major investment has been made in research to overcome the limits of both static and agitated cultures in order to achieve higher production rates, lower production costs, and/or shorter cultivation timeframes. One of the scale-up options that has been investigated is the design and development of efficient bioreactors [51]. Higher amounts of BNC production have been recorded in bioreactor cultures with continuous cultivation employing a spinning disc or airlift with continual oxygen delivery. BNC crystallinity, elasticity, and polymerization degree, on the other hand, were found to be lower than bacterial cellulose produced by agitated or static culture methods. Different bioreactor types, including as membrane [76], rotating disc [77], horizontal lift [9], and modified airlift [78] have been used to study the generation of BNC membranes or pellicles. The use of a stirred tank bioreactor can help to improve oxygen supply. However, this process uses a lot of energy. The airlift bioreactor is another popular form of fermentation reactor.

Chao et al. reported the first airlift bioreactor for BNC synthesis in 1997 [79]. In rotating disc bioreactor, numerous solids and fibers introduced directly into the medium and absorbed into the cellulose to enhance the characteristics of BNC and BNC-based biocomposites. The rotating disc bioreactor’s goal is to produce BNC with a uniform structure. Despite the fact that the BC produced in a spinning disc bioreactor is homogeneous, the outcome is not much higher than that of a static culture [80].

To create BNC pellets, fibrous material, or sphere-like particles, agitated culture bioreactors from the airlift [81] or modified airlift spherical bubble column reactor [82] types have also been tested. The bioreactor is combined with an enriched oxygen transfer capability in modified static bioreactors such as trickling bed reactors. The trickling bed reactor, in comparison to static culture and agitated/shaking systems, can deliver high oxygen concentration and minimal shear force. This bioreactor, which can generate high biomass density systems, can provide a higher surface-to-volume ratio than a static culture. BNC produced in a trickling bed reactor has a high polymerization, purity, porosity, water-holding capacity, high degree of OH association as well as and thermal stability [83].

Using a biofilm reactor, the production of BNC was successfully increased. According to one study, BNC production in a biofilm reactor was 2.5 times more than that obtained from static culture. The BNC had a higher crystallinity, a comparable crystal size, and better thermal performance than the static culture. Furthermore, mechanical strength study revealed that, like the pellicle form, the BNC produced restored tensile strength, a trait that could widen the BNC’s probable applications. Nevertheless, the water retention ability of a BNC generated in a suspended-cell reactor was lower [84].

Despite advancements in the industry, the use of bioreactors to generate BNC is currently limited, with most research being conducted on a small scale. Fundamental research of BNC biosynthesis remains critical in tandem with the development of innovative techniques using biotechnological reactors with enhanced process design. Cell-free extract technology is another technique for large scale production of BNC. A profound awareness of the biosynthetic route and characterization of functional cellulose synthase complexes would offer significant perceptions into the bioprocess and improve the efficacy of the BNC manufacturing process.

## 4. Application of bacterial nanocellulose

The demand of BNC for environmentally friendlier products is at its ever-increasing rate nowadays. BNC has unique mechanical and structural features including ultrafine fibrous networks, variable pore geometry, wider surface modification possibilities, well-suited to mold into different shapes, biocompatible, and biodegradable [3,85,86]. These characteristics made BNC as another wonderful biomaterial with breadth of applications in several fields [87,88]. BNC may well have applications in numerous fields, including food, paper, pharmaceuticals, nonwoven cloth, waste treatment, textile industry, refineries, and broadcasting, due to its unique properties, which include high density, purity, and degree of crystallinity, good shape retention, and higher surface area and water-binding capacity, when compared to native cellulose [85,87,89]. These unique characteristics have allowed it to be used in a variety of products, including high-grade papers, diet foods, tires, drug delivery systems, diapers, headphone membranes, textiles, high-performance sound systems and so on [48].

**Figure 5.**
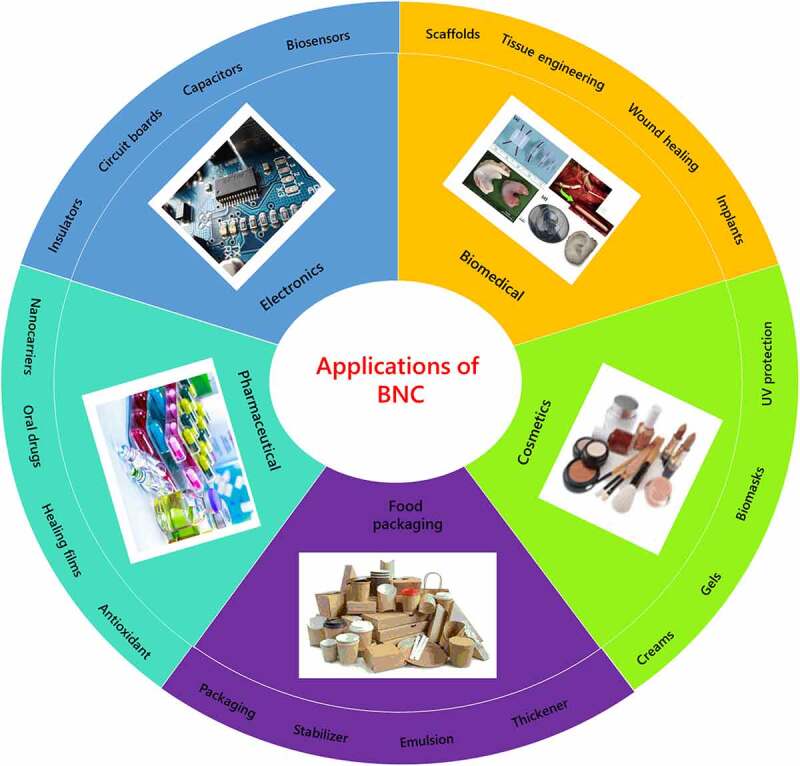
Applications of bacterial nanocellulose in different industries

Therefore, despite significant attempt to develop cost-effective BNC production methods by optimizing both downstream and upstream processes, scaling up cultivation remains a challenge, and the industry is still in the early stages of commercialization. When compared to traditional BNC fermentation processes, technology BNC manufacturing is extremely costly. Economic restrictions, including as costly capital investment, operating costs, and poor BNC productivity, are important bottlenecks to commercialization. Specific applications of BNC in different industrial sectors are described in Table 3. Also, recent advancements in the application of BNC in the food sector, personal care, biomedical, electronics, and so on are depicted in Figure 5.

**Table 3.** Specific applications of bacterial nanocellulose in different industries

Sl No.	Industry	Application	Reference
1	Food industry	Edible gels and coatings; ice-cream substituent to provide texture/structure modification and form stability; immobilization of probiotics and enzymes; food packaging	[48,87]
2	Personal care	Emulsion stabilizer (facial creams and masks); artificial nails; diapers; skin pigmentation agents; nutrient release agents	[107]
3	Biomedical	Biomedical clothing; dental implants; vascular implants; wound dressings	[90,119]
4	Electronics	Capacitors; flexible electrode materials; biosensors	[91,92]
5	Textiles	Materials with high absorbent capacity; tents and camping materials	[93,94]
6	Environmental protection	Ultra filtration membranes; sewage treatment; absorption of oil pollution, heavy metals and toxins	[95–98]
7	Paper industry	Paper with special characteristics; recycling used books; durable banknotes	[99,100]

### 4.1. Food industry

BNC is mostly utilized in food items as a stabilizing, gelling, and thickening agent, with its excellent water-holding capacity and nutritional fiber supplementation suggesting its usage in food products to enhance their quality [87,100]. Azeredo et al., (2019) reported the potentials of BNC as an ice-cream substituent for providing texture/structure modification, fat replacing and emulsion or form stability [86]. BNC was also reported for the immobilization of probiotics for controlling the gut microflora and control enzyme immobilization for providing health benefits in consumers These biomaterials can control the release of various enzymes namely laccase, lipase and lysozyme [101].

Silva et al., (2021) developed edible composite thin films from cashew apple juice derived BNC and cashew tree gum by combining in different proportions and a proportional increase in solubility, tensile strength, modulus, elasticity, and a decrease in permeability for the thin films were obtained using this BNC. The combination of two matrices facilitates to future food packaging’s with good thermal and mechanical stability [102]. Albuquerque et al. (2021) developed an active food packaging biocomposite using BNC/poly(3-hydroxybutyrate) system by activating with clove oil. The essential oil offered potential antimicrobial activity to the biocomposite blend and resulted increase in shelf-life by impeding microbial proliferation [103]. BNC products are categorized by US-FDA as GRAS products that can act as the supplement of dietary fibers in food. The major commercially available BNC products in food sector are Nata de coco (source: coconut) [104,105] and Nata de pina (source: pine apple) [106]. The different culture media and their sources mainly control the flavors of these food items. Cellulose-producing bacteria may be cultivated in culture media sources including fruit syrup, which allows the cellulose generated to take on the fruit’s natural taste and color [48]. Furthermore, BNC may be made in a number of forms and textures, allowing it to be used in a wide range of foods.

### 4.2. Personal care products

The quest of esthetics has resulted in a rising market for cosmetics as well as a wide range of personal care products. BNC as cosmetic additives improves transport of enriching nutrients by inducing easy penetration [107]. To produce the cosmetic products, natural polymers such as BNC in the form of pulp and hydrogels were employed as key ingredients. It has already been investigated as an agent for nutrient release, with promising results for the fast release of both hydrophobic and hydrophilic molecules.

Fernandes et al., (2021) reported the production of cosmetics using the blend of BNC and plant phenolics to cure the skin damages caused by irradiation of UV light [108]. BNC-based cosmetic masks such as ‘NanoMasque’ and ‘BioCellulose’ have been introduced in the recent decade in which BNC impregnated with active substances frequently used in cosmetics to induce biocompatibility. In Japan, BNC gels are commercialized as a face shield for personal care treatments [109].

Amorim et al. (2020) accounted the modification of properties of BNC using propolis extract in order to be utilized as an anti-infammatory sheet biomask providing long hydration to skin and infammations. The investigation supported the use of BNC as an ideal cosmeceutical biomaterial [6]. In order to produce innovative sustainable cosmeceutical solutions, recent advancements are leaning toward combining both dynamic ingredients and natural carriers. Stasiak-Rozanska and Ploska (2018) developed a feasible medium, corneum extract/BNC, for transferring 1,3-dihydroxy-2-propanone to induce skin pigmentation, which is a common skin abnormality that causes both mental and physical problems. The application of BNC adhesives in conjunction with 1,3-dihydroxy-2-propanone at a concentration of 50 g/L for 30 min produced skin that looked the closest to targeted natural tan. Unlike other commercial 1,3-dihydroxy-2-propanone-containing cosmetics, this formulation does not induce allergic reactions and may be used safely without leaving an unpleasant odor [110].

### 4.3. Biomedical fields

BNC finds immense applications in the biomedical field also. The ability to directly manage the biosynthesis process, as well as its high humidity control, biocompatibility, and non-toxicity, make membranes suitable for long term wound healing and burns. BNC is being explored for application in artificial blood arteries, heart valves, cartilage, and tissue engineering, in addition to dressings [87,88,111].

Cocarta et al. (2021) made mechanically improved BNC/methacrylate composites, with fine properties principally regulated by the degree of reinforcement and the amount of BNC in the composites. 2-Hydroxyethyl methacrylate hydrogels were chosen as a good monomer for hydrogel matrix due to their non-toxicity, biocompatibility, and wide, durable usage in biomedical applications. Therefore, the BNC/methacrylate are promising for tissue engineering, notably in tissue replacement, and wound healing [112].

Zmejkoski et al.(2018) developed a BNC/lignin composite hydrogel for the healing of chronic wounds. Clinically identified biofilm-forming bacteria *S. aureus, P. aeruginosa*, and *Serratia sp*., as well as laboratory strains *L. monocytogenes, S. aureus*, and *S. typhimurium*, were all inhibited by this combination. The composite was also shown a high ability for sustained release and dilatation of antibacterial chemicals that could be favorable to patients in terms of infection prevention and antimicrobial activity maintenance [113].

Chu et al. (2018) used a solvent exchange approach to make a fullerene water suspension. The fullerene was loaded into the BNC using the rehydration–dehydration technique. When exposed to light, these composites showed excellent antibacterial capabilities and a high rate of cell death, indicating that it might be used in multifunctional dressings for photodynamic therapy [114].

BioFill® was among the first commercialized product to meet the required specifications of an excellent wound dressing, such as better adherence to the wound, water vapor permeability, transparency, durability, elasticity, physical barrier for bacteria, low cost, easy handling, and hemostatic with minimal exchanges. Gengiflex® (for periodontal reconstruction), XCell®, and Bioprocess® are all BNC-based wound dressings that are at present existing on the market [115]. The healing process in chronic wounds using BNC dressing (DermafillTM, USA) revealed a reduced wound closure time from 315 days to 81 days, with 75% epithelialization of the afflicted areas [116]. A German group developed BASYC® (Bacterial Cellulose Synthetized), a tubular biomaterial for microsurgery of arteries and veins, with a focus on vascular applications [117]. Huang et al. (2021) used a silk fibroin-TEMPO oxidized BNC for high precision 3-D bioprinting of lung tissue scaffold [118]. Other potential BNC tissue engineering applications include ear cartilage, brain implants, urinary conduits, tympanic membrane, and vocal folds [119].

### 4.4. Electronic devices

Due to improvements in electrical technology, there has been a rise in demand for energy-storage devices that are both accessible and compact without compromising functionality. The usage of biodegradable polymers has gained a lot of interest due to the depletion of nonrenewable resources [100,120]. In solid-state electric double-layer capacitors, a novel gel electrolyte comprising 1-ethyl-3-methylimidazolium tetrafuoroborate and a separator was created utilizing BNC covered with chitosan and alginate layers. BNC inoculation, oxidation in a KIO_4_ solution, and coating with chitosan layers alternating with layers of alginate were used to make these gel electrolytes. The gel electrolyte was developed with the goal of being used in double-layered, solvent-free electric capacitors [120].

Luo et al., (2019) fabricated new cycled film-liquid interface culture methodology to develop ultra-strong, flexible, and highly conductive papers based on BNC and graphene [121]. Wan et al. (2018) investigated a synchronized deposition to form a ternary biocomposite composed of polyaniline/BNC/graphene nanosheets and the developed composite showed remarkable improvement in electrical conductivity. It was noted that this biocomposite can be utilized for designing of flexible electrode and electromagnetic shielding biomaterials [122]. Xie et al. (2018) also produced a biocompatible conductive material using BNC/polydopamine for developing flexible biosensors in wearable medical apparatus [123]. Huo et al., recently reported the designing of a simple and low-cost approach to produce 3D- surface-enhanced Raman scattering substrates using hybrid blending of Ag nanoparticles and BNC with outstanding stability, and signal reproducibility [124].

## 5. Future perspectives

BNC has attracted the interest of scientists and industrialists working in a variety of disciplines due to its unusual characteristics. However, the huge cost of production and genetic instability of certain *Komagataeibacter* species are significant barriers to their widespread use. It is hoped that genetic modification of BNC would make their applications more stable, efficient, and cost-effective than it was before. The rising variety of genome sequences and genetic tools available that could help researchers quickly identify the molecular components of BNC production and create rationally designed mutant strains. RNA-sequencing based on transcriptome analysis is an essential approach for identifying expression patterns in bacteria under various experimental circumstances.

Transcriptome studies, in contrast to genome analyses, focus just on transcribed genes that may be lead to more precise information about phenotype of *Komagataeibacter*. BNC with variable flexibility, mechanical characteristics, porosity or denser architecture according to the application can be designed using other innovative bacterial strains by genetically modifying bacteria will arise in near future. BNC is highly biocompatible to human cell lines, thus there could be more research possibilities on feasible genetic engineered strains to implement in biomedical and food industry. However, the majority of studies have used *G. xylinus*, and further research is desirable to investigate other bacterial strains can produce as much as *G. xylinus*. BNC productivity can also be enhanced by improving the cost-effectiveness of culture medium. Static fermentation necessitates more effort and time, resulting in a production capacity limitation. Several methods for increasing production efficiency include identification of high yielding BNC-producing strains, introduction of improved culture medium and bioreactors, and the use of automated equipment. Large-scale agitated fermentation can yield BNC, although non-cellulose mutations in bacteria restrict BNC productivity. As a result, BNC production capacities are constantly in need of improvement. If biotechnological techniques such as novel bioreactor design with optimized process parameters, and low-cost substrates in conjunction with high-yielding microbial species can improve undoubtedly the production of high-end nanocellulose.

This study shows that several BNC-based biocomposites created using various approaches are beneficial for hard tissue regeneration. Recently, manufacturing technologies such as bioprinting and 3D printing have shown tremendous promise in the development of 3D scaffolds for artificial biomedical devices. The gradual withdrawal of extremely complex BNC fibrils produced during biosynthesis is a key problem when employing BNC in 3D bioprinting. This nanofibril structure flocks and blocks the printer’s extrusion nozzle, preventing BNC from being used as bioink. As a result, more study on BNC-derived 3D printed biocomposites for the regeneration of hard tissue is required. Also, the development of BNC-derived bioinks for tissue regeneration is now in the laboratory stage, and significant experimental trials are required to move from manufacturing to effective clinical transfer.

As a result, advancements of BNC are possible, particularly in the biomedical industry as nanostructures for delivery of drug, cosmetics, and nutrients, and in environmental sectors to reduce organic pollutants or metals. Cell-free extract technology is only a developing technology that needs more researches to improve BNC production. BNC is a biomaterial of significant industrial importance and is recognized to have applications in diverse industries; further push is required to make this greener biomaterial a competitive product that is also economically feasible.

## 6. Conclusions

Many attempts have been made to make BNC production more practical by decreasing manufacturing costs, boosting yields, and improving overall performance by tailoring physico-chemical and biological characteristics. Metabolic engineering of microbes, exploring innovative strategies using bioreactors in both agitated and static modes, and replacing traditional cultural strains with cost-effective agricultural feedstocks to facilitate wider colonies of strains are three main approaches for increasing BNC production. BNC has a number of drawbacks, including low antibacterial action, poor processing solubility, and poor porosity. These drawbacks can be overcome by including bio-agents like as medicinal plant extracts, which can make BNC antimicrobial. Additional features like as cell adhesion, pore size, and porosity may be modified by combining BNC with a variety of different biopolymers. BNC might be used to build a range of biomedical scaffolds and personal care items if it can be produced in big numbers at an affordable cost.
